# Nanoconstructs for theranostic application in cancer: Challenges and strategies to enhance the delivery

**DOI:** 10.3389/fphar.2023.1101320

**Published:** 2023-03-15

**Authors:** Shivani Mishra, Tanvi Bhatt, Hitesh Kumar, Rupshee Jain, Satish Shilpi, Vikas Jain

**Affiliations:** ^1^ Department of Pharmaceutics, JSS College of Pharmacy, JSS Academy of Higher Education and Research, Mysuru, India; ^2^ Department of Pharmaceutical Chemistry, JSS College of Pharmacy, JSS Academy of Higher Education and Research, Mysuru, India; ^3^ Department of Pharmaceutics, School of Pharmaceutical and Populations Health Informatics, DIT University, Dehradun, India

**Keywords:** nanoconstructs, drug delivery, nanoparticles, theranostic application, cancer, autonomous delivery

## Abstract

Nanoconstructs are made up of nanoparticles and ligands, which can deliver the loaded cargo at the desired site of action. Various nanoparticulate platforms have been utilized for the preparation of nanoconstructs, which may serve both diagnostic as well as therapeutic purposes. Nanoconstructs are mostly used to overcome the limitations of cancer therapies, such as toxicity, nonspecific distribution of the drug, and uncontrolled release rate. The strategies employed during the design of nanoconstructs help improve the efficiency and specificity of loaded theranostic agents and make them a successful approach for cancer therapy. Nanoconstructs are designed with a sole purpose of targeting the requisite site, overcoming the barriers which hinders its right placement for desired benefit. Therefore, instead of classifying modes for delivery of nanoconstructs as actively or passively targeted systems, they are suitably classified as autonomous and nonautonomous types. At large, nanoconstructs offer numerous benefits, however they suffer from multiple challenges, too. Hence, to overcome such challenges computational modelling methods and artificial intelligence/machine learning processes are being explored. The current review provides an overview on attributes and applications offered by nanoconstructs as theranostic agent in cancer.

## 1 Introduction

Cancer is one of the major causes of mortality globally, and if a unique and adaptable anticancer platform is not created, it may account for 70% of all deaths over the next 2 decades (Dai et al., 2017). As per CA: A Cancer Journal for Clinicians, approximately 19.3 million cancer patients were diagnosed, and approximately 10 million deaths were reported in 2020 (Sung et al., 2021). It is estimated that cancer incidences will worsen in the upcoming years by increasing the number of cancer-related deaths to 13.1 million by 2030 (Chaturvedi et al., 2019).

Nanotechnology contributed enormously in cancer therapy and opens unique paradigm to address issues with existing chemotherapeutic agents (Bae et al., 2011). Nanomedicines have vast applicability in the diagnosis, and treatment of disease. It is a painless therapy that improves human health and can be used as a molecular tool for specialized medical interventions at the molecular level. Nanosystems have been used in the last 10 years for a variety of purposes, including drug supply (Goldberg et al., 2007; Ferrari, 2010), tissue regeneration (Goldberg et al., 2007), detection at the molecular level, molecular imaging (Godin et al., 2011; Hu et al., 2011), and direct therapy of various cancers, such as melanoma, lung cancer, and breast cancer (Gazeau et al., 2008; Mamo et al., 2010).

Theranosis is an important tool in efforts to apply precision medicine in clinical practice, which uses imaging agents to target diseases at the molecular level while combining therapy and diagnostics. Cancer theranosis is proven to be incredibly helpful for both doctors and patients. The basic objective of nanotechnology is to enable nanoparticle-based agents to efficiently and selectively distribute payload, while avoiding toxic manifestation, as well as to reliably track noninvasively delivered therapeutic efficacy over time. The designed theranostic agents may be stimuli-responsive, targeted, or nontargeted. Targeted theranostic carriers incorporate a targeting ligand that can adhere to the receptors overexpressed in tumors. Currently, nanoconstructs are a promising approach to cancer treatment, and they are generated by combining nanoparticles (NPs) with ligands. They have a simple design, geometry, and stability, that can be delivered either actively or passively. Nanoconstructs overcome the drawbacks of conventional cancer therapies, such as toxicity and untoward biodistribution. However, they suffer from some known limitations, such as biocompatibility, uneven distribution, toxicity and lack of precision. To address these issues, the concept of autonomous and nonautonomous drug delivery was introduced in which the blood flow parameters and tumor microenvironment (TME)-related factors were taken into consideration. This review covers the fundamental aspects of the importance, effect, and potential of nanoconstructs while focusing on the most recent advancements of nanoconstructs in cancer diagnosis and therapy.

## 2 Cancer: General perspectives

Cancer is a state in which a few cells of the body multiply uncontrollably and spread to other parts of the body. DNA damage, the major contributory factor for cancer, dysregulates various mechanisms that turn into specific malignant diseases. An unhealthy lifestyle and age has a significant influence on cancer occurrence (Wu et al., 2018; Quazi, 2022) Chikara et al. have reported that 19.3 million new cases of the cancer and more than 10 million deaths were reported in 2020, in worldwide (Chhikara and Parang, 2022). In the US, 1.9 million new cases and 609,360 deaths were reported in 2023 (Siegel et al., 2022).

Defective genetic changes or mutations in any of the listed genes, such as cytochrome P450 (CYP19, CYP2D6, CYP1A1), S-transferase (e.g., GSTM1, GSTP1), BRCA1/2, ATM, NBS1, PTEN, checkpoint kinase 2 (CHEK2), BRCA1 interacting protein (BRIP1), Nibrin (NBN), partner and localizer of BRCA2 (PALB2), RAD51C, RAD51D, malignant ovarian epithelial (MRE11A), Fanconi anemia, complementation group M (FANCM), tumor protein (p53), RAD50, BRCA1-associated ring domain (BARD1), alcohol, one-carbon metabolism genes (e.g., ADH1C, MTHFR), genes associated with DNA repair (XRCC1, XRCC3) and cell signaling receptors such as ER(Estrogen receptor), PR(Progesterone receptor), TNF-α(Tumor necrosis factor – α), may also contribute to the development of cancer (Easton et al., 2015; Walsh, 2015; Neidhardt et al., 2017). Furthermore, tumor heterogeneity, the TME, CSCs(Cancer stem cells), and epigenetics are factors responsible for the development of cancer, which may be accompanied by the development of chemotherapeutic resistance and recurrence associated with cancer (Jain et al., 2020).

The TME plays an important role in the development and poor prognosis of cancer (Baghban et al., 2020). The TME is highly heterogeneous in nature, and diverse cell populations make it more complex. The TME is highly associated with alteration or remodeling of the extracellular matrix (ECM), immune cells and other cellular processes in the development of drug resistance and relapse (Deepak et al., 2020). Tumor-associated markers such as tumor-infiltrating lymphocytes (TILs), tumor-associated macrophages (TAMs), cancer-associated fibroblasts (CAFs) and cancer-associated adipocytes (CAAs) are related to immune/tumor interactions, which contribute to the development of chemoresistance. Consistent tumor growth creates a hypoxic zone, acidic pH, and an anaerobic environment in the tumor mass (Lee and Griffiths, 2020). At the metastatic stage of cancer, chemotherapy is often not a promising therapy that will give a selective and broader response to patients (Jain et al., 2020).

Immunotherapeutic drugs have shown excellent growth by not only being the treatment for primary level cancer but also in avoiding metastasis and decreasing recurrence rates (Mahapatro and Singh, 2011). However, autoimmune problems are a main adverse effect of immune therapy. Furthermore, studies show that immune therapy is not as efficient for tumor cells as lymphoma (Kroemer and Zitvogel, 2018). A unique ECM is created by cancers that immune cells find difficult to infiltrate (Rosenberg et al., 2008). (Rosenberg et al., 2008). There are different types of immunotherapy for cancer, such as immune checkpoint inhibitors, T-cell transfer therapy, monoclonal antibodies, and vaccines. Immune checkpoints such as CLTA-4 and PD-L1 bind with other proteins of tumor cells and communicate with the immune system to allow for cancer development. Hence, various checkpoint inhibitors are used, such as PLGA-ICG-R837 for anti-CTLA4, and chimeric antigen receptor-mediated nanomedicines, which targets T cells (Cremolini et al., 2021).

## 3 Nanoconstructs

‘Nanoconstructs’ are two-component structures made of a ‘hard’ nanoparticle core and a ‘soft’ shell of biomolecular ligands that are often used for targeted applications drug delivery (Dam et al., 2014). Nanosystems contain the active cargo and carrier for the delivery of drugs, which are preserved, transported over biological barriers, and then released as payloads (Allen and Cullis, 2004). The primary benefits of nanoparticle-based chemotherapy are the rise in treatment specificity, increasing drug deposition at the targeted region, and lowering peripheral toxicity (Schmidt, 1990). The nanoconstructs should be designed in such a way that they can retain a variety of agents on the surface or inside its core to form an ideal tool for combination therapy. After the administration of nanoconstructs, their ultimate fate depends not only on the surface chemistry but also on the whole vasculature, which includes pressure, velocities, and heterogeneities in tissues. Hence, to determine whether the nanoconstructs reached the walls of vessels or persisted in blood, a computational modeling technique was used based on 4S parameters (size, shape, surface and stiffness) (Başağaoğlu et al., 2013; Coclite et al., 2017). Therefore, computational methods are used for complex phenomena, and designing optimal nanoconstructs for biomedical applications. (Cervadoro et al., 2018).

### 3.1 Computational modeling in nanoconstruct design

Computational models can be used to understand the complex processes associated with the TME, which play a crucial role in designing nanoconstructs. It helps in reducing the time and cost of production and helps in deciding the optimized dose. These models can demonstrate the distribution of drugs or theranostic agents inside the tumor, rate of flow, unbinding and binding of drugs to cancer cells, permeability, etc (Hubbard et al., 2017). These models determine the heterogenicity of cancer, interactions between cells, and cell behavior. They convert the hypotheses into mathematical rules and help in running the simulation experiments that discover the behavior of these converted hypotheses. There are various computational models used for cancer, such as lattice-based methods, off lattice methods, and boundary tracking models (Metzcar et al., 2019).

Cell-based computational models, also known as individual-based models or discrete models, are currently very popular for cancer; they simulate the cells that interact with virtual tissues and observe their single-cell behaviour and role in controlling cancer. Mathematical modeling helps provide valuable information about the tumor growth mechanisms and angiogenic process (Zangooei et al., 2021)**.** Hence, these models can be employed to understand the interaction between cancer and the therapeutic modality, which in turn will ease the design of optimal nanoconstructs for biomedical applications where experimental measurements are important (Cervadoro et al., 2018).

### 3.2 Building blocks for nanoconstructs

Nanoparticles (NPs) are known as particles with a diameter less than 1,000 nm and special characteristics that are often absent from major samples of similar types of material (Boisseau and Loubaton, 2011). All these can be categorized as 0D, 1D, 2D, or 3D depending on how the nanoparticle is shaped overall (Laurent et al., 2008). The layers of surface, shell, and core make up the primary components of NPs (Tiwari et al., 2012). They have become very popular in diverse disciplines because of their high surface to volume ratio, dissimilarity, submicron size, and better targeting abilities (Shin et al., 2016; Gavas et al., 2021).

Polymeric material-based nanoplatforms represent a class of widely used tuneable targeted delivery systems that can specifically deliver drug cargo to the desired site in a tumor. Nanoconstructs containing polymers such as poly(ε-caprolactone), (poly-l-lysine (PLL), poly(lactic-co-glycolic acid) (PLGA), and poly(propylene oxide) (PPO) are used for anticancer therapy. There are several advantages of using polymers, such as improved drug solubility, drug release, bioavailability, biodegradation, and reduced toxicity. Polymers for nanoconstructs are selected on the basis of target cancer cells, TME and toxicity status. Several lipid-based nanoplatforms are also being explored for the development of nanoconstructs (Alven and Aderibigbe, 2020)**.**

In addition to organic material-based nanoconstructs, inorganic nanoconstructs based on iron oxide, gold, silver and other nonmetallic materials have been explored for their potential therapeutic benefits. Among them, black phosphorus(BP) has become very popular in two-dimensional nanomaterials because of its special characteristics and structure. Its special features, such as biocompatibility and thermal, optical, electrical, and drug-loading characteristics, have increased its demand compared to graphene-containing 2D nanomaterials.

**FIGURE. 1:**
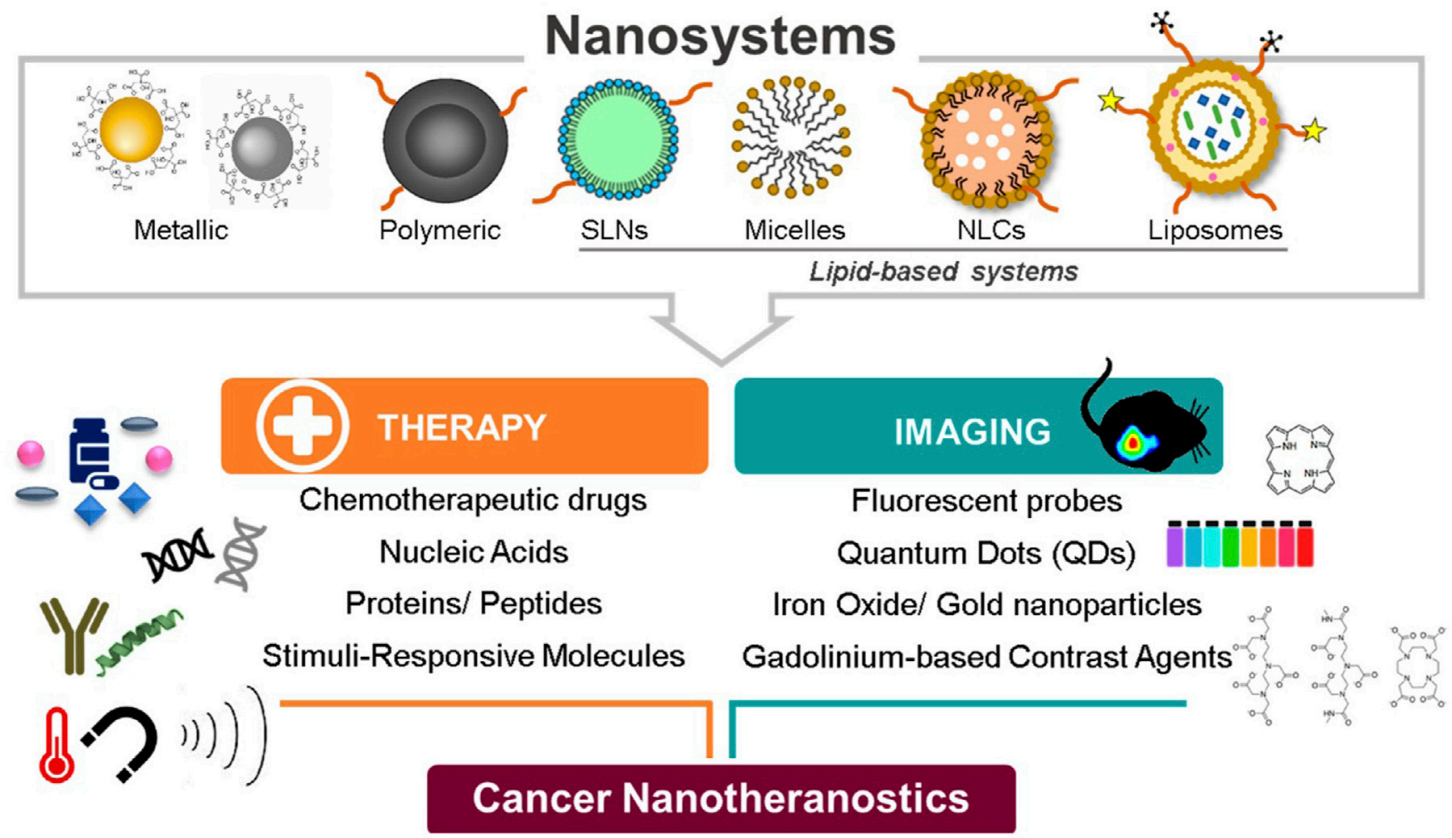
Nanotheranostics used in cancer therapy and imaging. Adapted from (Silva et al., 2019). © 2019 by the authors distributed under the terms and conditions of the Creative Commons Attribution (CC BY) license.

Due to the puckered honeycomb structure of BP nanomaterials, where each phosphorus atom is sp3 hybridized with a tetrahedral configuration, they exhibit exceptional optoelectronic, thermal, and mechanical capabilities. This gives them the ability to act as photothermal agents, which cause the photothermal ablation of tumors by converting NIR irradiation to heat. Additionally, the energy produced by the excitation of BP NPs can be transmitted to ambient oxygen and result in the formation of reactive oxygen species (ROS), which are crucial for the photodynamic therapy of tumors. Apart from the aforementioned materials used in the development of nanoconstructs, other organic NPs, inorganic NPs, and hybrid NPs are often utilized in the preparation of nanoconstructs. Representative examples of NPs are shown in Figure 1, and the approved marketed formulations used in cancer therapy are compiled in Table 1.

**TABLE 1:** Building blocks for nanoconstructs in cancer.

Carrier system	Material	Drug	Ligand	Indication	Reference
Polymeric nanoparticle	PLGA	Temozolomide	Cetuximab	For treatment of EGFR overexpressing cancers	Duwa et al. (2020)
	PLGA	ETP	LF	Enhanced anticancer activity in glioblastoma cells	Kuo and Chen (2015)
	PLGA	Docetaxel	Transferrin	Enhanced target selectivity and reduced toxicity in breast cancer cells	Cycle et al. (2019)
	PLGA	DTX	anti-EGFR antibody	Improved cytotoxicity and site specificity in non-small cell lung cancer	Patel et al. (2018a)
Bovine Nanoparticles	BSA	Rg5	FA	Breast cancer Therapy	Dong et al. (2019)
	BSA	Paclitaxel	HA	Ovarian cancer therapy	Edelman et al. (2017)
Silica or mesoporous silica nanoparticles	Silica	Paclitaxel	HA	Breast cancer therapy	Li et al. (2017)
	Mesoporous silica	DOX	HA	Enhanced targeting selectivity in HeLa cells	Palanikumar et al. (2018)
	Mesoporous Silica	Zinc complexes	Chitosan-Biotin	Enhanced chemotherapy	Kundu et al. (2022)
	Mesoporous Silica Nanoparticles	Epirubicin	GalNAc	Targeted cancer therapy of hepatocellular carcinoma	Cordeiro et al. (2022)
Dendrimer	PAMAM	miRNA	ferritin	Treatment of myeloid leukemia	Palombarini et al. (2021)
	Selenium	pDNA	FA	Cell specific targeting	Pillay et al. (2020)
SLN	Stearic acid	Curcumin	Transferrin	Prostate cancer therapy	Akanda et al. (2021)
		DOX	FA	Brain cancer therapy	Jain et al. (2022)
	Stearic acid	DOX	Peptide	Prostate cancer therapy	De (2021)
	Diacyl glyceride	Tamoxifen citrate	Transferrin	Breast Cancer Therapy	Bhagwat et al. (2020)
Carbon Nanotube	Multiwalled carbon nanotubes	DOX	FA	Breast cancer therapy	Omurtag Ozgen et al. (2020)
	Multiwalled carbon nanotubes	DOX	Carbohydrate galactose, mannose, and lactose	Breast cancer therapy	Thakur et al. (2022)
	Multiwalled carbon nanotubes	GEM	HA	Colon cancer therapy	Prajapati et al. (2019)
Liposomes	Phospholipids	DOX and SFB	Peptide	Breast cancer therapy	d’Avanzo et al. (2021)
	Phospholipids	AChE	Transferrin	Liver cancer therapy	Wang et al. (2021)
	Phospholipids	Paclitaxel	PCSK9	Targeted drug delivery to enhance anticancer activity in HEK293, HEPG2, HCT116	Charbe et al. (2022)
	Phospholipids	5-fluorouracil	FA	Targeted drug delivery to improve cytoxicity in HT-29, Caco-2, HeLa and MCF-7 cell lines	Handali et al. (2019)
Graphene oxide Nanoparticle	Graphene oxide	Metformin	HA	Triple negative breast cancer therapy	Basu et al. (2021)
	Monoolein	Paclitaxel	EGFR antibody fragment	Ovarian cancer Therapy	Zhai et al. (2018)
Metallic Nanoparticles	Monoolein	Copper acetylacetonate	HA	Selective targeting of CD44-expressing tumors	Pramanik et al. (2022)
	Gold	siRNA	FA	For gene silencing	Mbatha et al. (2019)
DNA nanorobot	DNA Origami	Blood coagulation protease thrombin	DNA aptamer	Cancer treatment for melanoma and ovarian cancer	Li et al. (2018)

Abbreviation- AChE-acetylcholinesterase; BSA-bovine serum albumin; DOX-doxorubicin; DTX-docetaxel; EGFR- epidermal growth factor receptor; FA-folic acid; GalNAc -triantennary N-acetylgalactosamine; GEM-gemcitabine; HA-hyaluronic acid; ICG-indocyanine green; LF-Lactoferrin; MSN- mesoporous silica nanoparticles; MWCNT- multiwalled carbon nanotubes; NPs-nanoparticles; PLGA-poly(lactic-co-glycolic acid); Rg5-Ginsenoside; SFB- sorafenib; SLN- solid lipid nanoparticle.

### 3.3 Cancer targeting through nanoconstructs

The development or engineering of a gene or drug release system with outstanding loading capacity to target tumors without affecting healthy cells is essential for effective cancer therapy. It is important to understand the targeting mechanisms and the cycle of interaction between cancer cells, NPs and tumors. Passive, active targeting, stimuli-responsive, and magnetic targeting are the few representative categories into which the targeting systems are divided.

#### 3.3.1 Passive targeting

**FIGURE. 2:**
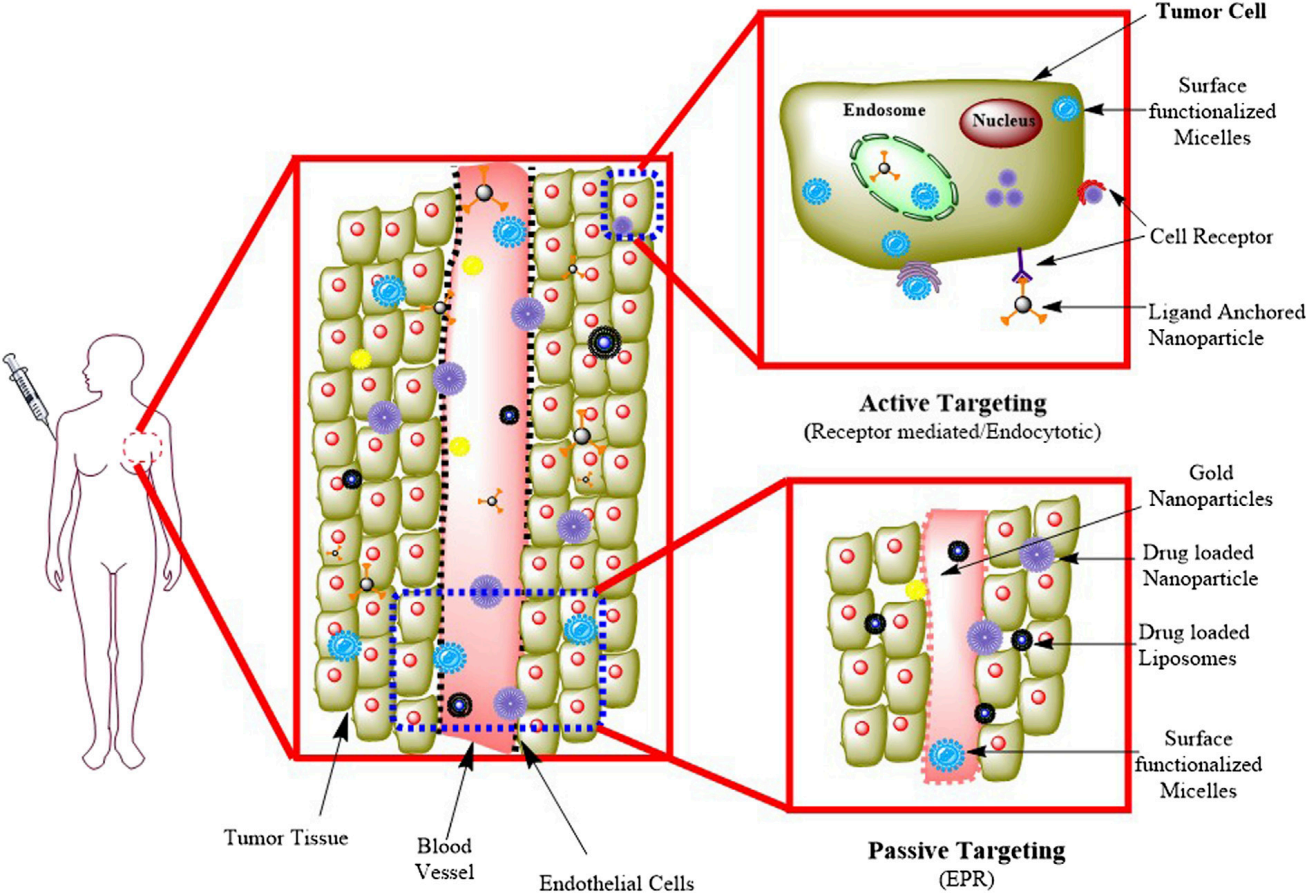
Passive and active targeting methods in drug delivery. Adapted from (Jain et al., 2020). © 2020 Elsevier B. V. All rights reserved.

The diffusion-mediated drug delivery method known as passive targeting includes the development of a complex of drug carriers. The complex of drug and carrier is transported to the target site *via* the bloodstream. Passive targeting nanoconstructs passively use the unique properties of solid tumors, such as a vasculature that is leaky and has reduced lymphatic clearance, to allow nanoconstructs to escape from the vasculature of the tumor and concentrate inside the tissues of the tumor *via* EPR (Black et al., 2014). For effective passive drug targeting, several characteristics, including molecular weight, surface charge, the surface’s nature, and its size, are important. For example, stealth liposomes coated with polyethylene glycol (PEG) will flow in the blood, and the surface charge on these liposomes has a major role in their longevity in blood circulation. PLGA-based NPs have the potential to load a high amount of drug and provide high specificity, stability, controlled drug release and a lower degradation rate (Rezvantalab et al., 2018). Polyvinyl alcohol (PVA) is also used for passive targeting, which helps in encapsulating hydrophobic drugs and their controlled release at specific tumor sites (Wei et al., 2020). The most often used method for the delivery of drugs in malignant cells is passive targeting, as shown in Figure 2.

#### 3.3.2 Active targeting

Active targeting is dependent on particular ligands or molecules, which bind to receptors or molecules that are particularly overexpressed on the target cells (Goldberg et al., 2013). This method of targeting is also known as ligand-mediated targeting (Ko et al., 2013). Here, NPs that contain ligands with specific properties, such as uptake and retention, must be in the target’s proximity to increase affinity. In active targeting, the nanoconstructs are delivered into the TME, which results in minimal damage to healthy cells. In this approach, strong binding ligands were used that bind with target receptors to initiate ligand‒receptor complex formation (Kue et al., 2016). In Figure 2, receptor-mediated endocytosis is explained, in which receptors such as folate and transferrin are present on the surface of the cell and capture specific target molecules or ligands such as proteins, peptides, and sugars. In this process, macromolecules are transported from the extracellular fluid. This approach helps enhance changes in the binding of NPs to cancer cells, improving drug penetration (Chaturvedi et al., 2019).

#### 3.3.3 Stimuli-responsive targeting

Cancer is characterized by the unique TME and abnormal cells and their complex mechanism. As discussed, conventional therapies for cancer have limitations because of nonspecific targeting and biodistribution. The performance of nanoconstructs can be affected by external stimuli (light, magnetic field, *etc.*) and internal stimuli (pH, enzymes, oxidative stress, *etc.*). During internal stimuli-responsive targeting, compounds such as calcium carbonate and glutathione are used with nanocarriers for pH-responsiveness. The responsiveness to pH/H_2_O_2_ was imparted to human serum albumin (HSA)-coated manganese dioxide (MnO_2_) NPs through albumin-based biomineralization of Mn2+. Generally, transducers convert external stimuli such as magnetic fields or phototherapy to physical quantities such as light radiation, which can be further converted into heat for effective cell killing (Fang et al., 2021).

In one of the published reports, magnetic field was utilized for the targeted delivery of the drug (Alexiou et al., 2011). In this system, superparamagnetic NPs were coated with chemotherapeutic agents and administered under the influence of a strong magnetic field to target the tumor. As a result, targeted delivery of the drug occurs in the tumor region. This technique is currently a promising approach because of fewer side effects and high specificity (Alexiou et al., 2011).

### 3.4 Ligands for forming nanoconstructs

Conventional chemotherapy without target selectivity usually produces significant side effects, reducing the effectiveness of a particular therapy. As a result, drug delivery methods need to ensure that they release drugs selectively and effectively up to the intracellular level at particular sites and result in low toxicity to maintain the quality of life of the patient. The appropriate choice of the ligands that may get adhered with particular receptors in cancer cells is one of the successful techniques for tumor-specific drug release. To design tumor-selective drug targeting systems, several ligands, such as hyaluronic acid, folic acid, peptides, and antibodies, have been widely utilized. To improve trafficking at the intracellular level for anticancer drugs, peptides or ligands that penetrate cells for tight junction valves are also used. In order to enhance the target selectivity and cellular uptake, different types of ligands are utilized in combination (Nguyen et al., 2021).

#### 3.4.1 Process for ligand selection

Delivering the therapeutic moiety with a specific ligand that has greater affinity to the pathological site is one of the best ways to enhance the effectiveness and safety of the drug. These types of systems offer advantages such as preventing toxicity to healthy cells and afford flexibility for optimizing the drug. Various ligands ranging from small molecules to aptamers and antibody fragments have been developed for this purpose. The application of ligand-targeted delivery systems extends to diagnosis as well. There are several factors that decide the selection of a targeting ligand. The ligand size that plays a role in pharmacokinetics of the drug and the ability of the ligand to bind to various receptor surfaces are crucial parameters. The binding affinity of the ligand influences the amount of the drug at the targeted site, while its chemistry can affect its binding capability with receptors. Immunogenicity, as in the cases of antibody‒drug conjugates, may affect the drug’s concentration and its half-life. Apart from this, selectivity of the target and other challenges, such as production cost and time, are the key criteria for ligand selection. Although there are techniques to overcome some challenges associated with ligands, for example, the stability can be increased by decorating the surfaces with PEG, these points should be keenly considered in the selection process of a ligand for achieving receptor-targeted drug delivery (Srinivasarao and Low, 2017).

## 4 Nanoconstructs in cancer theranosis

Cancer therapy is a novel intervention that offers potential benefits to both physicians and patients. This system incorporates diagnostic capabilities aligned with targeted delivery for real-time monitoring of cancer therapy (Ryu et al., 2012). Cancer theranosis depends on the depiction of different phenotypes at the cellular level detected by theranosis agent at the targeted tumor location, allowing for cancer therapy and observation of the theranostic compounds (Table 2).

**TABLE 2:** Nanoconstructs for theranostic application in cancer.

Carrier system	Material	Drug	Ligand	Indication	Reference
Polymeric Nanoparticles	PCL-PEG-PCL (PCEP)	DOX, ICG	FA	Breast cancer therapy	Hu et al. (2018)
	PLGA	SPIONs/DOX	AS1411 aptamer	Colon carcinoma	Mosafer et al. (2017)
	Poly (l-glutamic acid)	SPIONs/DOX	AS1411 DNA aptamer	Breast cancer	Hasannia et al. (2023)
	PLGA	I131	Anti-EpCAM	Breast Cancer, Osteosarcoma	Marshall et al. (2022)
Lipid Nanoparticles	DMKE	si RNA	anti-EGFR Aptamer	Gene delivery and bioimaging	Kim et al. (2017)
	Carnauba wax	SPIONs/DOX	Mannose	TNBC	Scialla et al. (2023)
Quantum dots	CdTe/CdS	-	FA	Breast cancer	Li et al. (2022)
	Graphene	-	HA	For targeted drug delivery and cancer cell imaging	Vahedi et al. (2022)
	CdSe/ZnS	DOX	Elacridar	Multi drug resistance cancer treatment	Wei et al. (2019)
Carbon nanotubes	Multiwalled carbon nanotubes	-	P-gp tagged antibody	Targeted photothermal therapy for cancer cells	Suo et al. (2018)
Graphene oxide Nanoparticles	Graphene oxide	DOX	GRPR-binding peptide	Glioblastoma therapy	Dash et al. (2021)
	Graphene oxide	Gastrin	Peptide	Near-infrared fluorescence imaging of oral squamous cell carcinoma	Li et al. (2020)
Lipid Micellar Nanoparticle	DSPE-mPEG	Paclitaxel, quantum dots	Anti-EGFR antibody	Colon cancer therapy	Kang et al. (2018)
Starch Nanoparticles	Starch	siRNA-IGF1R and DOX	Folate-biotin	Lung cancer Therapy	Li et al. (2019)
Nanoemulsion	Gadolinium	DTX	FA	Ovarian cancer Therapy	Patel et al. (2018b)
Metallic Nanoparticles	Gold		FA	Breast cancer and squamous carcinoma therapy	Mbatha et al. (2021)
	Gold		Triptorelin	Triple negative breast cancer therapy	Uzonwanne et al. (2022)
	SPIO	DOX	Glucose	Enhance antitumor efficacy on L929 cells	Thitichai et al. (2019)
	BPNS	DOX	HA	Breast cancer therapy	Peng et al. (2021)
	BPNS		HA	Metastatic breast cancer	Zhang et al. (2019)
	Gadolinium doped iron oxide	Polyethylenimine	Lenalidomide	Glioblastoma	Jani et al. (2021)
	BPNPs		FA	Breast cancer therapy	Deng et al. (2018)
	Zn(II)-dipicolylamine	-	cRGD	Breast cancer therapy	Zheng et al. (2019)
Dendrimer	PAMAM	Curcumin	MUC-1 aptamer	Colon adenocarcinoma	Alibolandi et al. (2018)
	PAMAM	DOX	F3 peptide	Triple-negative breast cancer	Yang et al. (2018)
	PAMAM	3,4-difluorobenzylidene-curcumin	FA	Ovarian and cervical cancer	Luong et al. (2017)

Abbreviation- BPNPs -black phosphorus nanoparticles; BPNS-black phosphorus nanosheets; BVA-bovine serum albumin; DMKE- O,O′- dimyristyl-N-lysyl glutamate DOX-doxorubicin; DTX-docetaxel; EGFR- epidermal growth factor receptor; FA-folic acid; GEM-gemcitabine; GRPR-gastrin-releasing peptide receptor; HA-hyaluronic acid; ICG-indocyanine green; NPs-nanoparticles; PLGA-poly(lactic-co-glycolic acid); SPIO- superparamagnetic iron oxide.

Nanotechnology has been identified as a possible option in cancer diagnosis and therapy. The majority of organic (polymer/lipids/dendrimers/liposomes, *etc.*) as well as inorganic NPs (metallic NPs/carbon nanotubes/quantum dots, *etc.*) imparted with imaging, treatment, and targeting abilities. The application of theranostic NPs in chemotherapy, photothermal therapy, siRNA/miRNA delivery, and other cancer treatments is currently the subject of substantial research.

### 4.1 Inorganic material based nanoconstructs

Black Phosphorus became very popular in two-dimensional nanomaterials because of its special characteristics and structure. To enhance the stability of these types of nanoconstructs, heterogeneous doping is used. BP can be conjugated with various types of metals, polymers, folic acid, albumin, *etc.*, and give theranostic effects in the biomedical field. Stimuli-responsive nanoconstructs can be developed to offer pH-mediated activation followed by NIR irradiation to offer stimuli-responsive BP-based anticancer therapy (Pandey et al., 2021). In one such application, the BP system has demonstrated its potential use in gene delivery. Mcl-1 belongs to Bcl-2 group and is a possible target for cancer therapy. Breast cancer cells also showed Mcl-1 amplification. BP nanomaterials conjugated with PLL for Cas13a/crRNA delivery were created to target Mcl-1 transcription. *In vitro* experiments on AGS cells revealed a 58.64% decrease in Mcl-1 expression as well as a suppression of cell activity (Schacter et al., 2014).

In addition to magnetic resonance imaging (MRI), optical imaging methods have been used for these nanoparticulate systems. Because of their bright fluorescence and great photostability, quantum dots have been frequently utilized in nanoparticulate systems to examine particle transport *in vitro* and *in vivo* (Bae et al., 2011).

Among the stimuli-responsive systems, the pH-responsive system carries a great advantage of modifying the delivery system as per the internal environment. One example is mesoporous silica nanoparticles (MSNs), which are novel pH-responsive drug delivery systems where the drug is attached to the surface via covalent bonds, which are pH-responsive linkages. These are a class of upconversion NPs that are a special type of optical nanomaterial doped with lanthanide ions and exhibit a wide range of electronic transitions in the 4f electron shell. These NPs have the ability to upconvert two or more photons of lower energy into one photon of higher energy. The nanoconstruct is prepared using copper ions and metal-phenolic networks of tannic acid on the surface of mesoporous silica-coated upconversion NPs. Real-time monitoring is possible using these systems with simultaneous drug release at the target site. Anticancer drugs have been incorporated into these systems; thus, this nanoparticulate form helps in carrying out efficient cancer therapy (Hu et al., 2019).

Moreover, for better cancer imaging and therapy, nanoconstructs are prepared with copper sulphide (CuS) NPs on the surface of mesoporous silica nanoshells containing porphyrin molecules and labelled with [^89^Zr]. These hybrid nanoconstructs were biocompatible, enhanced tumor deposition, and increased blood retention time. It can help in tetramodal imaging during cancer therapy with complete tumor elimination without any side effects. It is an efficient approach to combine imaging and therapeutic benefits in a single nanoconstruct (Goel et al., 2018).

Hyaluronic acid, as a targeting moiety for CD44 receptor-overexpressing cancer cells, works very well for controlling tumor growth and alterations. The trio-responsive chemo-phototherapy nanoconstruct includes various agents such as chemotherapeutic drug (DOX), duo-photothermal agents such as CuS and graphene oxide, and hyaluronic acid as the targeting moiety. The nanoconstruct shows proper drug release and increased photothermal properties with efficient ROS generation as the three major responses. High deposition and retention of nanoconstructs is seen in tumors, which is reflected by the photothermal and biodistribution profile. These types of nanoconstructs inhibit tumor growth by analyzing and identifying the tumor volume, apoptosis, and proliferation speed. Thus, these nanoconstructs show great potential in the translational cancer nanomedicine field (Poudel et al., 2020).

Targeted alpha therapy is another useful radiotherapeutic technique that can be used as a promising treatment against cancer. The main limitations of this technique are the conjugation of radionuclides with vectors and confinement of the dose. In this study, silica NPs were conjugated with transferrin and a chelator and loaded with ^225^Ac. The resultant nanoconstructs were highly and selectively cytotoxic and facilitated better excretion and minimal deposition in bones (Pallares et al., 2020).

Dam et al. (2014) reported an improvement in the *in vitro* efficiency of nanoconstructs containing gold by increasing the loading of G-quadruplex aptamers, which shows how citrate-type buffers at low pH can be used to load the outer surface of gold nanostars (AuNSs) with oligonucleotides and DNA aptamers (Dam et al., 2014).

### 4.2 Polymer based nanoconstructs

Currently, polymeric nanoconstructs are being employed for cancer theranosis because of the flexibility associated with their surface modification, stimuli responsiveness and ability to incorporate hydrophilic as well as lipophilic bioactives or diagnostic agents. One such example is deformable discoidal nanoconstructs, which are used as a novel delivery approach for imaging and therapeutic purposes. These are made by the polymerization of PEG and PLGA in a discoidal shape. These polymer matrices include hydrophobic and hydrophilic microdomains that act as pockets for various imaging and therapeutic compounds. These particles reduce rapid sequestration from the Mononuclear phagocyte system (MPS) by circulating in the bloodstream for a longer duration. Additionally, these polymeric matrices can easily integrate contrast agents, lipid-drug conjugates, and polymer-drug conjugates, leading to the creation of true theranosis agents (Palange et al., 2017).

Currently, fluorescence resonance energy transfer (FRET) is a novel optical imaging modality that may be used to monitor drug release from NPs at the targeted tumor location (Caldorera-Moore et al., 2011). In this regard, doxorubicin(DOX)-loaded polyethylene glycol-block-peptide (FFKY)-block-tetraphenylethylene (PEG-Pep-TPE/DOX) NPs were developed and monitored for changes in FRET signals in A549 cells. Synergism was observed between DOX and the self-assembled peptide with real-time observation of drug release from the developed system (Wang et al., 2020).

In addition, PLGA-based nanoconstructs were explored to offer radiodynamic therapy. This anticancer therapy is based on the production of ROS at the tumor site. It mainly works on the hypoxia caused by the tumor, due to which a reduction in oxygen levels results in the production of ROS. There is one novel approach in which nanoconstructs are made of PLGA NPs that are loaded with verteporfin and perfluorooctylbromide. These nanoconstructs, when placed under normoxic and hypoxic conditions, show a sudden increase in ROS production. This therapy has killed ∼60% of pancreatic cancer cells in humans and suppressed the growth of tumors within 2 weeks. These success rates show that nanoconstructs based on radiodynamic therapy provide better and non-invasive treatment for deeply located hypoxic tumor (Clement et al., 2021).

Other polymeric nanoconstructs, such as polyurethanes (PU) nanoconstructs, are widely used for biomedical applications because they are part of the stimuli-responsive and biodegradable material class. PU nanoconstructs are simple delivery systems for drug and cancer treatment. These types of nanoconstructs have various types of properties, such as rapid drug release, the solubility of hydrophobic-type chemo drugs, targeting, improving efficiency, and stimuli sensitivity. They can be conjugated with ligands for active targeting. They are sensitive to pH, temperature, stimuli, and various external factors; hence, all these factors make them perfect nanocarriers (Gajbhiye et al., 2020).

Among lipid-based nanoconstructs, PEGylated squalene (SQ-PEG)-based nanoconstructs were also used for cancer therapy. They assemble with lipophilic pyropheophorbide-a (Ppa) and form nanoconstructs, with an average size of 200 nm and drug loading capacity of 18% (w/w). These nanoconstructs show 99.99% fluorescence quenching. Its bioavailability and phototoxicity can be identified through light eradication *in vivo*. These nanoconstructs show good diagnostic potential when tested in the chick embryo model implanted with U87MG glioblastoma microtumors (Adriouach et al., 2019).

Apart from polymer- and lipid-based nanoconstructs, dendrimer-based nanoconstructs offer multiple benefits as theranostic agents. Dendrimers made up of poly(amidoamine) (PAMAM) have been widely used for cancer nanomedicine applications and are a family of synthetic macromolecules that contain various types of functional groups and well-organized interior structures. First-generation dendrimers have one main limitation of their small size, which causes limited capacity for drug loading, and resist passive targeting for a tumor because of increased retention time and permeability. They are also not stimuli-responsive nanoconstructs, so to overcome all these limitations, superstructured dendrimeric nanoconstructs (SDNs) were generated.

### 4.3 Dendrimer based nanoconstructs

PAMAM dendrimers are spherical, highly branching macromolecules that can stabilize metal NPs such as gold NPs while encapsulating active compounds. A study explored the theranosis potential of curcumin-loaded dendrimer-gold hybrid structures. By combining generation five poly(amidoamine) dendrimers with PEGylated amine-terminated AuCl_4_^−^ ions, a dendrimer-gold hybrid structure was developed. The final hybrid system was loaded with curcumin attached to the MUC-1 aptamer. The results showed increased cellular cytotoxicity in HT29 and C26 cells in comparison to the nontargeted system and established potential in both cancer therapy and CT scan-based tumor imaging (Alibolandi et al., 2018).

Another study demonstrated the chemical production of unimolecular micelle-based hyperbranched PAMAM dendrimers conjugated with F3 peptide to target cellular nucleolin overexpressed in MDA-MB-231 cells. PAMAM micelles with F3 attachment (PAMAM-DOX-F3) demonstrated better uptake in MDA-MB-231 cells. For PET imaging, the ^64^Cu was chelated to micelles to monitor their pharmacokinetic behaviour. Serial PET imaging revealed that ^64^Cu-PAMAM-DOX-F3 accumulated in MDA-MB-231 tumors quickly, effectively, and persistently compared to ^64^Cu-PAMAM-DOX. The distribution characteristics in other organs and tissues were remarkably comparable (Yang et al., 2018).

### 4.4 Miscellaneous nanoconstruct

The number of approved therapies that use the simultaneous intake of two or more pharmacological therapeutic compounds or a combination of different treatment approaches has progressively expanded (Gane et al., 2017). Often, even the most potent drug molecule may not be enough to completely cure the problem. Because of this, the modern administration of two or more therapeutic agents may aid in synergistically achieving more intracellular targets and destroying the targets more efficiently (Gee et al., 2005). One such example is the drug fumagillin, which was administered as a single injection of αVβ3 integrin-targeted paramagnetic NPs when given in combination with oral atorvastatin in experimental rabbits, where antiangiogenic activity was observed with a prolonged effect (Winter et al., 2008). In another study, protein-based hybrid NPs encapsulating docetaxel (DTX) and poly (sodium-4-styrenesulfonate)/DOX -modified gold nanorods were developed for combined plasmonic-based photothermal therapy (PPTT) and dual-chemotherapy. The release of DOX was controlled by NIR radiation, while DTX was released following diffusion. The cytotoxicity results in MDA-MB-231 cells demonstrated synergism between the two drugs followed by NIR irradiation (Villar-Alvarez et al., 2019). In one of the reports, a polyvalent theranostic nanocarrier was developed with a core made of superparamagnetic iron oxide nanoparticles (SPIONs) and a surface made of folic acid-polyamidoamine dendrimers (FA-PAMAM). To increase its solubility and evaluate its therapeutic potential, a very effective hydrophobic anticancer drug called 3,4-difluorobenzylidene-curcumin (CDF) was also coloaded in the FA-PAMAM dendrimer. Targeted NPs that were produced as a result (SPIONs@FA-PAMAM-CDF) have strong MR contrast and better anticancer activity on SKOV3 and HeLa cancer cells (Luong et al., 2017).

## 5 Challenges in the use of nanoconstructs in cancer therapy

**FIGURE. 3:**
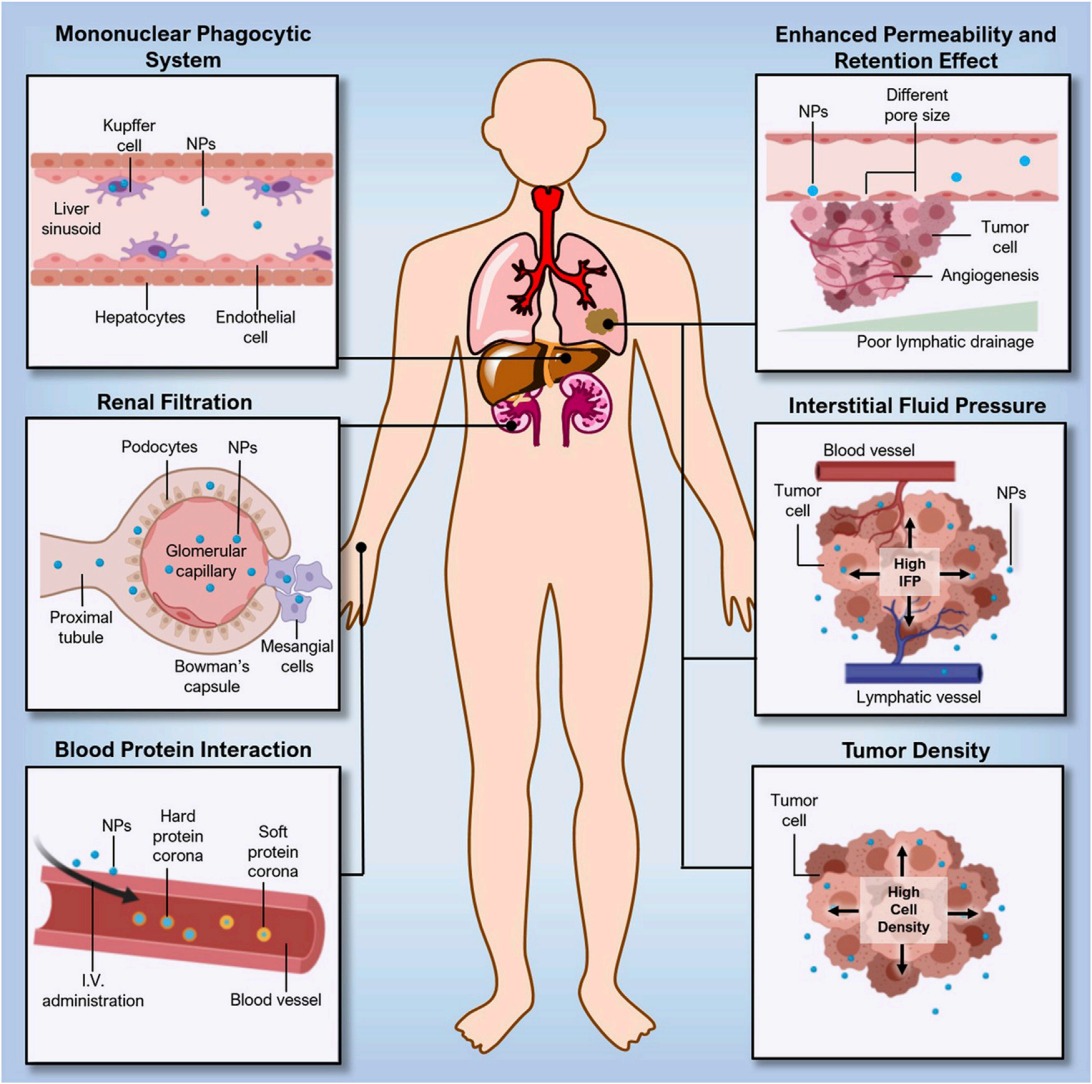
Challenges in the delivery of nanoconstructs. Adapted from (Saw et al., 2021) © 2021 Wiley-VCH GmbH distributed under the terms and conditions of the Creative Commons Attribution (CC BY) license.

The Enhanced Permeability and Retention Effect (EPR) effect is a process that is explained by the hyperpermeation and extended retention of biomolecules encapsulated in nanocarriers, such as lipoproteins, hormones, and albumins, retained in solid tumors which contains leaky vasculature, elevated endothelial macromolecule transcytosis, and a lack of functional lymphatic drainage inside its interstitium (Figure 3).

To obtain EPR effect-based selective tumor deposition, low extravasation of normal-type tissue and renal clearance are needed. The nanoconstructs’ reduced particle size, which is lower than tumour vascular permeability, allowed them to easily pass through and produce the EPR effect (600–800 nm). However, the nanoconstructs have the bigger particle size than the pore of blood capillary (6–12 nm) and renal filtration (5–6 nm), influences the targeting of NPs (Choi et al., 2009).

Multiple tumors may develop tumor interstitium with different ECM (e.g., fibrin, fibronectin, hyaluronan collagen, and proteoglycans), tumor parenchyma and stroma cell compositions due to variations in the tumor type and stage. The variations in physical rigidity and pressure ranges may affect the distribution and transport of nanoconstructs in the tumor, which may further affect their therapeutic efficacy (Swartz and Lund, 2012; Perry et al., 2017). Hepatocellular carcinoma, Kaposi sarcoma, renal cell carcinoma, and cancer of the neck and head (Regev et al., 2005) are known to be high-level EPR tumors, whereas pancreatic ductal carcinoma and prostate cancer (Maeda, 2015) are referred to as low-EPR tumors.

There are other factors that hinder nanoconstructs distribution and deposition in tumors other than drawbacks of the EPR effect, such as biological and physical hurdles in delivering an adequate dosage of a therapeutic drug to the targeted site of the tumor. (Bae and Park, 2011). After intravenous delivery, nanoconstructs undergo multiple events, such as adsorption of proteins, diffusion of particles, aggregation, shear force destruction and hydrolysis. (Florence, 2012; Lazarovits et al., 2015). These types of events influence the number of nanoconstructs that eventually reach the tumor. The degree to which these events impact nanoconstructs tumor accumulation may vary depending on the physicochemical characteristics of the nanoconstructs (Gould et al., 2015). Another challenge is incorrect perceptions derived from the selection of *in vivo* models at the preclinical stage. *In vivo* studies performed on animal-derived cancer models do not show a resemblance to human cancers. The ratio of a person’s body weight to their tumor’s size is comparatively lower than those in animal models. As a result, it is not a surprise that the amount of nanoconstructs reaching the tumor sites in humans is probably less than the requisite therapeutic range.

## 6 Approaches to enhance the delivery of nanoconstructs to the cancer site

Despite the issues outlined above, several ways to address these problems may facilitate nanoconstructs penetration and deposition into tumors. A generic classification adapted to facilitate drug release and enhance the effectiveness of nanoconstructs comprises autonomous and nonautonomous systems of drug delivery.

Nonautonomous cancer treatment was classified as actively and passively aimed nanoconstructs. The modification of TME with the help of NPs can be considered a promising approach for the management of cancer (Jia et al., 2021). It is an established fact that cancer cells survive in an oxygen-deficient environment known as a hypoxic environment. The integral switch to switch off mitochondrial energy production is independent of ATP and oxygen supply and switches on the alternate pathways to produce energy in oxygen-deficient environments. Thus, by stopping the cells from switching on the alternate pathway, cancer growth can be controlled. To make this possible, many NPs are coming into play. For example, the mitochondrial chaperone TRAP-1 is structurally and functionally similar to the Hsp90 family of proteins, which have the potential to switch on alternate pathways for energy production in tumor cells. Hence, nanocarriers to deliver TRAP-1 inhibitors were developed in which iron oxide nanoparticles (IONs) were conjugated to the Hsp90 inhibitor geldanamycin (GA) and the mitochondria localization signal (MLS) peptide to enable selective tumor targeting (Amash et al., 2020). One of the recent studies examined the impact of the surface curvature of nanoconstructs on endosomal pathways using CpG (cytosine-phosphate-guanine)-conjugated spiky and spherical gold NPs. Administration of spherical NPs followed by spiky NPs prompted the production of larger late-stage endosomes. The outcomes from this research demonstrate a possible impact of nanoconstructs design on intracellular fate (Lee et al., 2022).

**FIGURE. 4:**
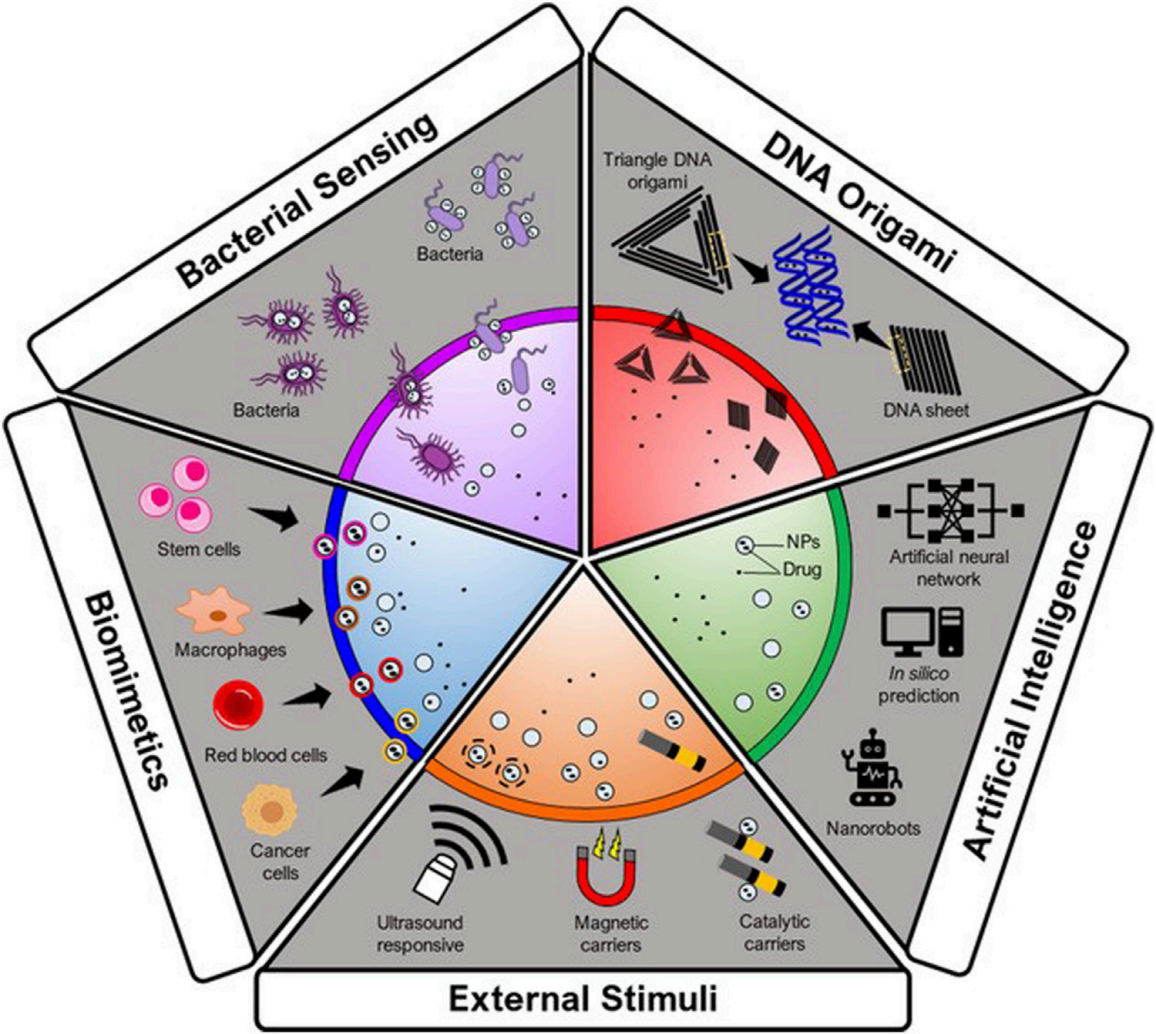
Autonomous delivery systems. Adapted from (Saw et al., 2021) © 2021 Wiley-VCH GmbH distributed under the terms and conditions of the Creative Commons Attribution (CC BY) license.

The autonomous delivery systems are described as ‘therapeutic missiles’, as they can transport the nanoconstructs to the target regardless of the flow of blood and its direction. Recent advancements in autonomous-type delivery systems indicate that biomimetic-type delivery and biohybrid bacteria have the desired therapeutic potential (Yu et al., 2020). In one report, a nanomedicine-loaded bacterium that can propel itself was used to cure cancer. This autonomous swimmer destructs itself and delivers the anticancer medication at the targeted site (Figure 4). Incubation and electroporation were two methods that were utilized for introducing NPs into the bacteria, where incubation was found to be less effective than electroporation. More precisely, DOX-containing 100-nm liposomes were injected into motile bacteria (*Salmonella*) (Zoaby et al., 2017). When the bacteria enter cancer cells, the drug is internally released to kill the cancer cell. *Salmonella* was chosen over *E. coli* to build the nanoswimmer platform due to its higher velocity in the TME and its capacity to target tumors and penetrate triple-negative cancer cells. In general, the TME favours bacterial motility, which is dependent on glucose and pH (Pontier-Bres et al., 2012).

The sciences of nanomedicine and artificial intelligence are two highly powerful tools for advancing the cause of personalized medicine. Drug synergy is a constant problem for cancer patients receiving any type of drug administration because it depends on time, dose, and patient at any point in the therapeutic process. Furthermore, due to significant intratumor and interpatient heterogeneities, it is difficult to rationally design diagnostic and therapeutic delivery systems and study their outcomes. Closing these gaps and improving the accuracy of diagnosis, drug delivery, and therapy requires the integration of artificial intelligence technologies (especially data mining, neural networks, and machine learning) (Soltani et al., 2021).

An innovative statistical method for developing controlled release drug delivery systems(CRDDS) is the artificial neural network (ANN). When there is no obvious functional dependence between the inputs and outputs, it is one of the best methods to be relied upon. ANNs can also be used to model complex biological data and nonlinear systems (Cheung and Rubin, 2021). Other uses for ANNs include cancer classification, predicting protein secondary structures, and solving multiresponse and multivariate system problems (Bi et al., 2019). The relationship between process variables, formulation, and CRDDS drug release profiles is not implicit or linear. As a result, related networks can be used in conjunction with other ANN model types. These ANN models can be used to depict the relationship between process variables, formulation, and response, such *as in vitro* drug release patterns (Patel and Patel, 2016). In comparison to conventional therapies, the application of AI (Artificial Intelligence) in the formulation of nanotheranosis can be a great benefit for better positioning of diagnostic and therapeutic substances into the body. If imaging agents and medications need to be loaded into a certain carrier, predictive AI algorithms can be utilized to forecast encapsulation efficiency (EE%) (Soltani et al., 2021). For instance, a QSPR model was used to predict, with greater than 90% accuracy, whether molecules may be loaded into the carrier based on the circumstances of the encapsulation process and their chemical makeup. A similar method can be employed to evaluate the cytotoxicity of other NPs and to assess the impact of surface modification on biocompatibility. When talking about medical imaging, it is important to consider how AI can contribute to image analysis. By using the aforementioned methods in imaging from nanos, one can better comprehend the therapeutic efficacies and particle biological dispersion patterns (Xu et al., 2019).

Currently, Nanorobotics is the emerging technology of constructing robots in the nanometric scale, which are creating a niche in the biomedical field (Raaja et al., 2016). In similar line, DNA based nanomaterials controlled by aptamer encoded logic gate can be utilized as autonomous delivery systems (Douglas et al., 2012). Since DNA is a natural substrate for computing, it has benefitted a diverse set of logic circuits and robotics (Katsnelson, 2012). In this context, DNA origami can be utilized to develop nanorobots which can intercommunicate and can be activated to release the drug cargo at the targeted site. This will allow the real time monitoring, faster delivery and computer assisted drug delivery (Khulbe, 2014; Devasena Umai et al., 2018).

There are multiple other examples in which DNA nanorobots can target HER2-positive breast cancer cells to induce apoptosis (Ma et al., 2019), deliver drug cargo to the cancer tissue mediated by nucleolin targeted therapy (Li et al., 2018), identification of cancer biomarkers (Mirzaiebadizi et al., 2022) and act as biosensors to allow the detection of target oligonucleotides and miRNAs (Domljanovic et al., 2022).

## 7 Image-guided drug delivery systems for cancer therapy

Conventional drug delivery systems for anticancer therapy frequently lack the ability to target medications to certain organs or tissues of interest (tumors), as well as the ability to track or image the *in vivo* fate and assess the effectiveness of drug administration. Imaging tumor regression in patients after targeted therapy X-ray, CT or radiography, will be considered a separate intervention altogether. Multiple objectives can be accomplished with a single dose by co-administering the image guided molecules incorporated in the delivery system. It may facilitate evaluation of extent of drug targeting, sites of localization, excretion, and imaging (Iyer et al., 2012).

In one of the reports, PSMA aptamer-conjugated DOX-loaded iron oxide NPs were developed that could be used as theranostic agents in prostate cancer. These systems detect prostate cancer sites (by MRI) and even deliver the drug DOX to the target tissue (Yu et al., 2011).

Treatments that comprise biocompatible NPs could provide precise optical/MR imaging and treatment to cells that overexpress folate receptors. The co-encapsulation of DTX and NIR dyes into NPs, which are novel additions to nano delivery systems for the detection, diagnosis, and treatment of malignancies, was also produced using a modified solvent diffusion approach (Santra et al., 2009).

In one such approach, light-activated theranostic NPs made up of PEG-modified polyacrylamide combined with iron oxide NPs and photofrin, a strong photosensitizer. were used for the imaging and photodynamic treatment of brain malignancies., F3 peptide was used as a ligand to specifically target nucleolin receptors present on endothelial cells and tumor cells. This system showed antitumor effects in a rat brain tumor model for brain targeting (Reddy et al., 2006).

Another method relies on bifluorescence resonance energy transfer (Bi-FRET) technology, which was applied to quantum dot (QD)-aptamer conjugates for simultaneous tumor imaging and DOX delivery. A functional RNA aptamer was attached to the surface of quantum dots and DOX was embedded in the aptamer., By triggering the fluorescence of QDs, this nanosystem was able to visualize drug delivery as well as to target prostate cancer cells (Bagalkot et al., 2007).

In continuation, pH-responsive polymeric micelles were developed with an aim of *in vivo* imaging and photodynamic therapy of tumors (Zhong et al., 2019), where, methoxy PEG was conjugated with a pH-sensitive polymer, poly (b-amino ester). Self-assembly of the block copolymer with protoporphyrin IX, a radiosensitizer, led to the formation of nanomicelles. pH-responsive release of protoporphyrin IX was observed in the acidic environment of tumors. Clear tumor accumulation and complete tumor ablation were observed by fluorescence imaging using tumor-bearing mouse models. Thus, these systems show great potential as photo dynamic theranosis (Koo et al., 2010).

## 8 Conclusion and future prospectives

Nanoconstructs designed for theranostic applications in cancer offer improved detection, precise delivery of drug cargo to tumors and fewer devastating effects on healthy organs. Additionally, imaging approaches can evaluate the effectiveness of medications in real time by using customized probes. Understanding the TME and cancer-associated events with the help of computational modeling will enable the development of nanoconstructs with desired attributes. A plethora of nanotechnology-based platforms have been explored for theranostic applications, including nanomaterials from organic and inorganic origins. Exogenous and endogenous stimuli play vital roles in controlling drug release and augmenting the autonomous and nonautonomous mechanisms of targetability. The full potential of DNA origami, nanorobots, artificial intelligence and machine learning has yet to be explored for the development of nanoconstructs for cancer theranosis. Although enormous progress has been made in the development of nanoconstructs for theranostic application, very few of them could advance to the clinical phase of investigation. However, the solution always lies in advanced nanomaterials, which will open up a new vista for the development of multimodal NPs equipped with better diagnostic and therapeutic capabilities. The desired outcomes through the advent of nanotechnology can only be facilitated by conducting multidisciplinary studies in a large cohort of patients.

## Glossary

AChE: Acetylcholinesterase
AuNSs: Gold nanostars
ANN: Artificial neural network
AI: Artificial intelligence
BP: Black phosphorus
CAAs: Cancer-associated adipocytes
CAFs: Cancer-associated fibroblasts
CDF: 3,4-difluorobenzylidene-curcumin
CSCs: Cancer stem cells
CuS: Copper sulfide
DOX: Doxorubicin
DTX: Docetaxel
ECM: Extracellular matrix
EPR: Enhanced permeability and retention
ER: Estrogen receptor
FA: Folic acid
FRET: Fluorescence resonance energy transfer
GA: Geldanamycin
HSA: Human serum albumin
IONs: Iron oxide nanoparticles
MLS: Mitochondria localization signal
MnO2: Manganese dioxide
MPS: Mononuclear phagocyte system
MSNs: Mesoporous silica nanoparticles
NPs: Nanoparticles
PAMAM: Poly(amidoamine)
PEG: Polyethylene glycol
PLGA: Poly(lactic-***co***-glycolic acid)
PLL: Poly-l-lysine
Ppa: Pyropheophorbide-a
PPO: Poly (propylene oxide)
PPTT: Plasmonic-based photothermal therapy
PR: Progesterone receptor
PU: Polyurethanes
PVA: Polyvinyl alcohol
ROS: Reactive oxygen species
SDNs: Structured dendrimeric nanoconstructs
SPIONs: Superparamagnetic iron oxide nanoparticles
SQ-PEG: PEGylated squalene
TAMs: Tumor-associated macrophages
TILs: Tumor-infiltrating lymphocytes
TME: Tumor microenvironment
TNF-α: Tumor necrosis factor-alpha
TPE: Tetraphenylethylene
CRDDS: Controlled release drug delivery system
QD: Quantum dot
ICG: Indocyanine green
EGFR: Epidermal growth factor receptor
Rg5: Ginsenoside Rg5
BSA: Bovine serum albumin
HA: Hyaluronic acid
LF: Lactoferrin
SLN: Solid lipid nanoparticle
MWCNT: Multiwalled Carbon Nanotubes
GEM: Gemcitabine
SFB: Sorafenib
GalNAc: Triantennary N-acetylgalactosamine
GRPR: Gastrin-releasing peptide receptor
BPNS: Black phosphorus nanosheets;
BPNPs: Black phosphorus nanoparticles
DMKE: O,O′- dimyristyl-N-lysyl glutamate
